# Supplementary material for: Uptake-Dependent and -Independent Effects of Fibroblasts-Derived Extracellular Vesicles on Bone Marrow Endothelial Cells from Patients with Multiple Myeloma: Therapeutic and Clinical Implications
Source: Biomedicines. 2023 May 8;11(5):1400. doi: 10.3390/biomedicines11051400 (PMC10216205; doi:10.3390/biomedicines11051400)
Supplement: Supplementary file 1 [file biomedicines-11-01400-s001.zip › biomedicines-2173640-supplementary.pdf]

Supplementary Figures

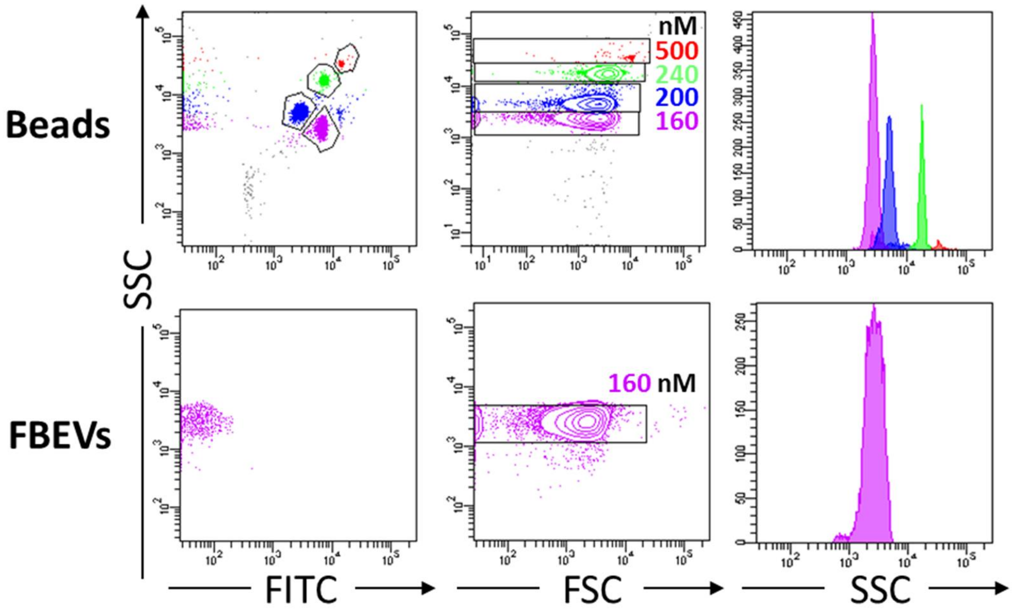

Supplementary Figure S1. FBEVs characterization by flow cytometry. Comparison of flow cytometry analysis of FBEVs *versus* FITC-labelled beads to assess size of EVs based on forward scatter count (FSC) and side scatter count (SSC) distribution. Note that distribution of FBEVs falls in the 160 nm region. A representative dot plot analysis is shown.

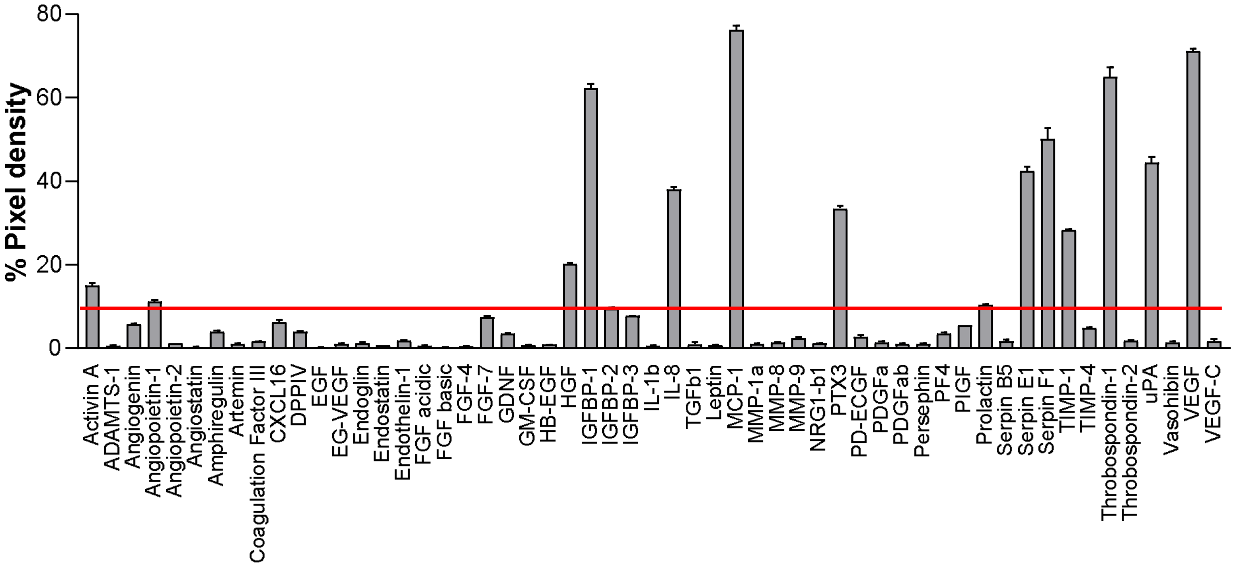

Supplementary Figure S2. FBEVs contain a pro-angiogenic cargo. FBEVs were lysed and analyzed for cytokine cargo by an angiogenic array. Data have been normalized to reference spots and expressed as mean Pixel Density ± S.D. The red line marks the 10% pixel density cutoff and was used to facilitate data visualization.
